# What is the evidence for the management of patients along the pathway from the emergency department to acute admission to reduce unplanned attendance and admission? An evidence synthesis

**DOI:** 10.1186/s12913-017-2299-8

**Published:** 2017-05-16

**Authors:** Sarah H. Credé, Colin O’Keeffe, Suzanne Mason, Anthea Sutton, Emma Howe, Susan J. Croft, Mike Whiteside

**Affiliations:** 1 School of Health and Related Research (ScHARR), The University of Sheffield, Sheffield, England; 2 Sheffield Teaching Hospitals NHS Foundation Trust, Sheffield, England; 3 Doncaster and Bassetlaw Hospitals NHS Foundation Trust, Doncaster, England; 4 School of Health and Related Research (ScHARR), The University of Sheffield, Regent Court, Regent Street, Sheffield, S1 4DA UK

**Keywords:** Emergency medicine, Unplanned attendance, Avoidable admissions

## Abstract

**Background:**

Globally, the rate of emergency hospital admissions is increasing. However, little evidence exists to inform the development of interventions to reduce unplanned Emergency Department (ED) attendances and hospital admissions. The objective of this evidence synthesis was to review the evidence for interventions, conducted during the patient’s journey through the ED or acute care setting, to manage people with an exacerbation of a medical condition to reduce unplanned emergency hospital attendance and admissions.

**Methods:**

A rapid evidence synthesis, using a systematic literature search, was undertaken in the electronic data bases of MEDLINE, EMBASE, CINAHL, the Cochrane Library and Web of Science, for the years 2000–2014. Evidence included in this review was restricted to Randomised Controlled Trials (RCTs) and observational studies (with a control arm) reported in peer-reviewed journals. Studies evaluating interventions for patients with an acute exacerbation of a medical condition in the ED or acute care setting which reported at least one outcome related to ED attendance or unplanned admission were included.

**Results:**

Thirty papers met our inclusion criteria: 19 intervention studies (14 RCTs) and 11 controlled observational studies. Sixteen studies were set in the ED and 14 were conducted in an acute setting. Two studies (one RCT), set in the ED were effective in reducing ED attendance and hospital admission. Both of these interventions were initiated in the ED and included a post-discharge community component. Paradoxically 3 ED initiated interventions showed an increase in ED re-attendance. Six studies (1 RCT) set in acute care settings were effective in reducing: hospital admission, ED re-attendance or re-admission (two in an observation ward, one in an ED assessment unit and three in which the intervention was conducted within 72 h of admission).

**Conclusions:**

There is no clear evidence that specific interventions along the patient journey from ED arrival to 72 h after admission benefit ED re-attendance or readmission. Interventions targeted at high-risk patients, particularly the elderly, may reduce ED utilization and warrant future research. Some interventions showing effectiveness in reducing unplanned ED attendances and admissions are delivered by appropriately trained personnel in an environment that allows sufficient time to assess and manage patients.

## Background

The year-on-year increase in emergency hospital admissions creates additional pressure on health systems internationally and is a trend that is not abating. In the last 15 years these admissions have increased in England by 47% [1]. Admission rates are known to vary widely between healthcare systems [2], the majority of this variation is explained by unemployment rates and urban/rural status, however some variation is explained by factors that are modifiable within healthcare services [3]. Healthcare service related factors associated with higher rates of potentially avoidable admissions included those related to the patient pathway from the emergency department (ED) to acute admission, i.e. ED attendance rate, the conversion rate of ED attendances to admissions as well as the proportion of short stay admissions [3]. Short stay admissions are often managed in designated assessment or observation wards/units to reduce crowding in EDs and avoid unplanned admissions [4].

A previous review suggests that there is insufficient evidence for interventions that reduce unplanned hospital admission in secondary emergency and acute settings [5]. Patients arriving in the ED will typically be assessed, managed, discharged home or admitted to hospital. Prior to admission to a hospital ward, this pathway may also involve assessment and management in an acute medical unit, typically for 24 to 72 h [4]. Along this patient journey surprisingly little evidence exists to inform the development of interventions to reduce unplanned hospital admissions and attendances at the ED. Management within the ED, acute assessment and observation units is key in establishing how to optimise care to reduce unnecessary variation in emergency admissions across urgent care systems.

This study reviewed the evidence on interventions to manage people with medical presentations, including those with long-term conditions and the frail elderly, who present with an acute event to reduce unplanned emergency hospital attendance and admissions. This review focusses specifically on the patient pathway from the ED to admission, including the observation ward or acute assessment unit, and uses ED attendance, re-attendance, as well as hospital admission as primary outcome measures.

## Methods

A rapid evidence synthesis, using a systematic literature search, was undertaken. The search was further enhanced by supplementary search methods. As this was an evidence synthesis, following systematic review methodology, ethical approval and consent were not required.

### Search strategy

Electronic data bases of MEDLINE, EMBASE, CINAHL; The Cochrane Library and Web of Science were searched, using a pre-determined search strategy, for the years 2000 – current (2014). Search terms relating to emergency medical services or acute care, medical assessment or clinical decision units, avoidable admissions or re-attendance, demand/burden on health services, chronic disease, long-term conditions, comorbidities, and the aged, were combined into a single search strategy which was translated across the five bibliographic databases listed above. Searches were limited to all adults (16 plus years) and English language publications only. Comments, letters and editorials were excluded as publication types from the search. Supplementary searches included citation searching of key references and a thorough review of reference lists of included papers and published reviews. Experts within the field of emergency and acute medicine were also consulted for additional references.

### Selection criteria

Evidence included in this review was restricted to controlled and observational studies in peer-reviewed journals. Articles reporting on interventions to reduce unnecessary or avoidable unplanned ED/hospital care in emergency departments and acute medical units or acute care settings were included. Acute medical units receive patients from emergency departments for expedited specialist assessment and treatment for a period of 24–72 h before discharge or ward transfer [4]. As not all hospitals have acute medical units it was decided that any study reporting an intervention that began within 72 h of ED attendance or hospital admission would be included. Acknowledging that many interventions occur along the patient’s clinical pathway and include important assessments before, and patient management after, the attendance we included interventions that occur within the ED, acute medical units or acute care settings or those that span these settings.

To be eligible for inclusion the study needed to report at least one outcome related to attendance at the ED, re-attendance or unplanned admission to hospital. These outcomes did not have to be the primary outcome of the studies to be eligible for inclusion; although in some papers they will have been. Where the primary outcome was to reduce admissions or re-attendance this is indicated in the summary Tables 1 and 2. The definitions of the study outcomes provided by the study authors were, in many instances, insufficient to determine whether re-attendances or readmissions included all presentations, for any presenting condition, within the defined time period or only those for the same unresolved problem. Every attempt was made to identify whether re-attendances and readmissions were related to the original episode of care.

**Table 1:** Summary table of studies describing interventions based in the Emergency Department

Study (Author, Year, Country)	Target population	Study Design	Intervention	Control	Outcomes	Results/Main Findings	Quality
Emergency Department (ED) based interventions (during ed attendance)							
Specialist aged care pharmacist							
Mortimer et al., 2011, Australia [12]	Patients: ≥ 65 years with chronic condition or ≥70 years without a chronic condition, all with Australian Triage Category classification >1 (do not require immediate medical attention).	Non-randomised study, alternative allocation based on time of presentation and availability of practitioner. All patients initially assessed by ED doctor.	Medication reconciliation and review, patient education by specialist aged care pharmacist (ACP) and referral where necessary (n = 101). Patients admitted or discharged from Emergency Medicine (EM) department.	Usual-care review by ED doctor (n = 98). Patients admitted or discharged from EM department.	Proportion of patients re-presenting (with the same unresolved problem) to hospital within 14 and 28 days.	No significant difference between the proportions of intervention and control patients re-presenting to hospital within 14 and 28 days. Intervention group patients had a significantly greater average ‘length of stay’ in the Department of Emergency Medicine compared with the control group patients (12 hours : 42 minutes, n = 101 vs. 10 hours : 05 minutes, n = 98, respectively, P < 0.01). Reduced admission rates for intervention group 73/101 vs 92/98 control group (not tested for significance).	Non-randomised study. Potential selection bias, pilot study only.
Patient education in the ED							
Smith et al., 2008, Australia [14]	Adult patients, >18 years, arriving at the ED with an acute exacerbation of asthma (diagnosed prior to this visit). Patients excluded if too ill or require intensive medical treatment.	RCT, 2 inner city EDs.	Patient centred education (PCE) underpinned with learner centred principles. Patient had to prioritise the 6 asthma curriculum steps according to perceived need, patients then educated accordingly. Education given during ED presentation (n = 68).	Standard patient education. Following steps 1 to 6 (sequentially) through curriculum (n = 78).	ED secondary care re-attendance rates at 4 and 12 months.	No significant difference between groups at 4 months OR 0.4, (95% CI 0.2 -1.1. No significant difference in re-attendance at 12 months (p = 0.96). In the sub-group of patients with no prior GP care, the PCE patients had significantly fewer re-attendances at 4 months OR 0.1 95% CI 0.0-0.7) and 12 months OR 0.2 95% CI 0.0-0.6. In subset of patients discharged from ED: PCE group had significantly fewer re-attendances at 4 and 12 months OR 0.3 95% CI 0.1-0.9 and 0.3 95% CI 0.1-0.8.	Single researcher educated all patients. Possible contamination of control group patients admitted (may have received further education in hospital).
ED initiated interventions with community component							
Comprehensive Geriatric Assessment							
Mion et al., 2003, USA [26]	Patients ≥ 65 years, community-residing and fit for discharge (selected from two EDs). Patients designated either high or low risk for repeat ED attendance, hospitalization or nursing home placement and randomisation within each risk status group.	RCT. (2 EDs) Block randomisation based on stratification by risk of re-attendance.	Comprehensive geriatric assessment in ED by advanced practice nurse & referral to community/social agency, primary care or geriatric clinic. Follow up by nurse after visit by telephone to confirm contact with follow up physician (n = 324).	Usual care (any referral recommendations to community responsibility of participant or proxy to follow up) (n = 326).	Subsequent ED visits at 30 and 120 days, hospitalization at 30 and 120 days after index visit.	No statistically significant effect on overall service use rates at 30 or 120 days. Sub-group analysis by risk classification at triage. Among the low-risk patients usual care patients less likely to return to the ED in first 30 days than intervention group patients OR 1.9 95% CI 1.0-3.5. No difference in low risk group at 120 days or in high-risk group at 30 or 120 days.	Sample size did not reach the recruitment goal of 800.
McCusker et al., 2003, Canada [30]	ED patients aged ≥ 65, ready for discharge from ED without further intervention but identified as at risk of subsequent ED attendance on Identification of Seniors At Risk (ISAR) questionnaire.	RCT, multisite (4 EDs).	Geriatric nursing assessment in ED using standardized checklist. Referrals to community health centre, primary physician or other community service where appropriate were made by ED nurse (n = 166).	Usual care (n = 179).	Return visits to ED in month after ED visit.	Intervention group patients more likely to make a return visit to the ED OR 1.6 (95% CI 1.0 to 2.6). Excess ED visits in intervention group limited to patients who hadn’t visited their physician before the index ED visit.	ED staff not blinded to intervention. Individuals not randomised (day of week randomised). Nearly a fifth of patients randomised to intervention group were not able to receive intervention.
Caplan et al., 2004, Australia [9]	Community dwelling older people (≥75 years) discharged home from single urban ED.	RCT (18 month follow up).	Comprehensive geriatric assessment (CGA) over a four week period. CGA would involve any assessment by a specialist nurse who initiated urgent interventions and care plan in ED. Consultation between nurse and inter-disciplinary team including geriatrician weekly led to any further intervention/referral to appropriate practitioner (n = 369).	Usual discharge plan by medical team. (n = 369).	Primary: admissions to any hospital within 30 days of the initial ED visit. Secondary: elective and emergency admissions.	At 18 months significant difference in the rate of emergency admissions in favour of intervention (44.4% vs 54.3%; p = .007). At 30 days after the initial ED visit significantly fewer total admissions (elective and emergency) in the intervention group than in the control group (61 intervention (16.5%); 82 control (22.2%); p = 0 .048. Although no significant difference in number of emergency admissions at 30 days (P = 0.312). No significant difference in visits to ED (without admission) within 30 days (p = 0.349)	Assessments post intervention not blinded. Some control group patients may have had CGA from another service.
Arendts et al., 2012, Australia [7]	Patients ≥65 yrs presenting to two EDs with one of the ten presenting complaints often resulting in admission (UTI, respiratory tract infection, fall with minor injury, hip/knee pain, back pain, heart failure, angina, syncope, TIA, new confusion/delirium). Patients requiring urgent medical treatment were excluded.	Non-randomised controlled clinical trial. (2 EDs)	Early comprehensive input from allied health (care coordination team (CCT)) prior to discharge. CCT team included physiotherapist, occupational therapist and social worker. Physician (usually a geriatrician or geriatric trainee), nursing and other allied health staff such as speech therapists were co-opted to assist the teams as required (n = 3165).	Usual pre-discharge assessment (n = 2100).	Primary outcome: Admission to an inpatient bed from the ED.	Unadjusted 2.4% absolute reduction in admissions in the intervention group. Adjusting for non-randomised design and patient factors the reduction in admissions overall was non-significant (OR 0.88, 95% CI 0.76-1.00, p 0.046). Adjusted sub-group analysis showed significant differences in admissions favouring intervention for angina OR = 0.71 (0.53-0.93) and musculoskeletal OR = 0.67 (0.49-0.93).	Non-randomised study. No follow up of short term readmissions in either group.
Arendts et al., 2013, Australia [8]	Community dwelling patients (≥65 yrs) attending 2 EDs with non-emergency problem. Patients screened at initial assessment to identify any risk (e.g. falls risk, impaired living) associated with early discharge and assigned to cases or controls based on ‘risk’ or ‘no risk’.	Non-randomised controlled study (2 EDs). Patients identified as those fit for discharge from ED and underwent discharge risk screening. Positive screen formed the intervention group and matched with controls that were identified as ‘low risk’ on risk screen.	Input from a care coordination team (CCT) prior to discharge for patients screened as at risk from discharge. CCT team included physiotherapist, occupational therapist and social worker. Physician (usually a geriatrician or geriatric trainee), nursing and other allied health staff such as speech therapists were co-opted to assist the teams as required (n = 1098).	Usual assessment for patients in ‘no risk’ from early discharge (n = 1098).	Primary outcome measure: unplanned ED re-attendance within 28 days.	Unadjusted difference of 3% in 28 day unplanned ED re-attendance rates (17.9% cases, 14.8% controls, *P* = 0.05). At 1 year 43.4% of cases and 29.5% of controls had experienced at least one unplanned hospitalisation (P < 0.001).	Non-randomised study. Differences in outcomes unadjusted. Patients in two groups at different risk from discharge.
Foo et al., 2014, Singapore [32]	Patients ≥ 65 years with a TRST (triage risk screening tool) score of 2 or more and who were planned for discharge.	Quasi-randomised controlled trial.	Risk stratification and focused geriatric screening by Geriatric Emergency Medicine nurse. Focused areas included cognition, mood, continence, visual acuity and hearing, mobility and social issues. Medication reconciliation and postural blood pressure undertaken. Intervention and referral (e.g. geriatric assessment clinic, post-acute home care) and discharge education provided where appropriate (n = 569).	Standard ED care (n = 587).	ED re-attendance and hospitalisation.	The reduction in ED re-attendance (OR 0.75, CI 0.55-1.03, p = 0.07) and hospitalization (OR 0.77, CI 0.57-1.04, p = 0.09) were not significant.	Non-randomised study; large percentage of eligible patients refused to take part or had left ED prior to being asked to take part.
Multi-factorial falls intervention							
Shaw et al., 2003, UK [21]	Patients ≥65 years, cognitively impaired or with dementia, referred after fall. Mini-mental state examination score <24. Exclusions medical diagnosis causing fall such as CVA, unable to walk.	RCT (2 EDs within same NHS trust.)	Multifactorial intervention initiated in ED. Multifactorial clinical assessment (Medical, cardiovascular, physio, OT) followed by intervention for all identified falls risk factors (n = 130).	Assessment followed by conventional care (n = 144).	Fall-related attendances to A&E and fall related admissions.	No significant differences between groups for fall related attendances to A&E (OR 1.25; 95% CI: 0.91 to 1.72), fall related admissions and mortality (OR 1.11; 95% CI 0.61 to 2.00).	Small trial, single trust. Limited blinding, for certain outcome measurements only.
Davison et al., 2005, UK [16]	Patients ≥65 years presenting to ED with fall or fall-related injury and at least one additional fall in the preceding year.	RCT (2 EDs in a university teaching hospital and an associated district hospital).	Multifactorial medical and falls assessment including fall history, cardiovascular assessment, gait and mobility assessed by physio and assessment of home risk by OT. Intervention initiated in ED and continued at home by physio/OT where necessary (n = 159).	Usual care provided by ED and primary care physicians (n = 154).	Fall-related hospital admissions and ED attendance over 12 months.	No significant differences in falls related ED attendance (RRR 0.90; CI: 0.55–1.47) or fall-related hospital admission (RR 0.80; CI: 0.41–1.56).	Relatively small sample size of 313, only 282 of patients remained in study at the end of year. There was lack of comparative data on fall risk factors in the control population.
Specialist nurse assessment in ED							
Hegney et al., 2006, Australia [10]	Patients >70 years presenting to ED. Patients readmitted for renal dialysis, chemotherapy, palliative care or mental health reasons; and patients from high care residential care facilities excluded.	Before and after study.	Specialist community nurse in the ED undertaking a risk-screening assessment using Screening Tool for Elderly People (STEPS) prior to discharge. Referred to Home and Community Care Service co-ordination team (or direct to community provider) if necessary. (n = 2139).	Before and after design.	Primary outcomes: re-presentation (patients who had previously presented to the ED within the last seven days with same presenting problem) and readmissions to the ED.	Re-presentation rates at the end of the post-intervention period 16% lower than the rates prior to the start of the intervention (X ^2^ = 15.59, P < 0.001) Readmission rates at the end of the post-intervention period were 5.5% lower than the rates prior to the start of the intervention (X ^2^ = 4.61, P < 0.05).	Before and after study design. Differences in service use in intervention period may have been due to seasonal effect in demand.
Nobel et al., 2014, UK [18]	Adults ≥ 18 attending the ED for established epilepsy (documented diagnosis ≥1 year).	Prospective, non-randomised intervention study. (3 EDs).	Epilepsy nurse specialist self-management intervention. Patients offered 2 one-to-one sessions with epilepsy nurse specialists (ENS) and treatment as usual. Recruited in one ED and intervention on out-patient basis (n = 44).	Recruited from 2 EDs. Treatment as usual (n = 41).	Epilepsy-related ED use 12 months post recruitment.	No significant effect on ED visits at 12 months. OR 1.92 (95% CI 0.68, 5.41).	Non-randomised intervention. Low recruitment rate of eligible patients.
ED initiated discharge interventions (discharged directly from ED)							
Personal emergency response systems (PERS)							
Lee et al., 2007, Canada [29]	Patients ≥70 who presented to single urban ED after a fall identified as fit for discharge to own home. Patients recruited in ED or within 72 hours of discharge home.	RCT (Single blind).	Conventional discharge planning plus free use of personal emergency response systems (PERS). PERS could be triggered by patient in an emergency and directed them to central monitoring station for assessment of response required (e.g. neighbour/relative or 911) (n = 43).	Conventional discharge planning (included assessment by Geriatric Emergency Nurse) (n = 43).	Return visits to the ED within one year of index visit to ED.	Return to ED within 60 days occurred in eight of 43 patients in both the control and treatment groups (RD, 0.0%; 95% CI −16% to 16%). Hospitalization occurred in six of 43 in the control group versus three of 43 in the treatment group (RD 7.0%; 95% CI −19.8% to 5.9%).	Small RCT examining short term impact only. Selection bias by patients refusing to participate or withdrawing.
Nurse led telephone/telehealth post discharge intervention							
Biese et al., 2014, USA [22]	Patients aged ≥ 65 discharged to own home from ED with instruction to seek outpatient follow-up.	RCT (single ED).	Post discharge telephone call–mediated intervention by a nurse at 1 to 3 days after each patient’s index ED visit to review discharge instructions and check compliance with medication and/or physician follow up (n = 39).	Placebo group- call to assess patient satisfaction with care (n = 35). Control group - no follow up (n = 46).	Secondary outcome:. Probability of return visit to the ED within 35 days of the index ED visits.	No differences in ED visits or hospital admissions within 35 days of discharge from the ED (p = 0.41).	Small sample size 160 initially, final analysis (120). Study not powered to identify a decrease in return visits to the ED.
Wong et al., 2004, China [36]	All patients (adults and children) presenting to ED with problems related to fever, respiratory or gastrointestinal condition. Discharged home from ED and contactable by phone after discharge.	RCT (single ED at acute general hospital).	Two follow up calls from an ER nurse 1–2 days and 3–5 days **after ER discharge** (n = 395).	Usual post-discharge care (n = 400).	30 day ER return visits.	Significant difference in ER revisit within 30 days. (p = 0.036). Intervention group more likely to return within 30 days.	A number of children included in this study.
Guttman et al., 2004, Canada [28]	Patients aged ≥ 75 years discharged from ED who reside in private home or residence and contactable for follow-up telephone interviews.	Pre/post study. Pre (standard discharge care). Post (intervention - nurse discharge plan coordinator)	Nurse discharge plan coordinator (NDPC) - patient education, coordination of appointments, telephone follow-up and access to NDPC for 7 days after discharge (n = 819).	Standard discharge care (n = 905).	Unscheduled revisits to the ED within 14 days of the index visit.	Non-significant reduction in relative risk of unscheduled return visits in first 14 days for NDPC group (unadjusted RR 0.79; 95% CI 0.62 - 1.02.) Adjusted for severity of illness significant reduction in unscheduled return visits at day 14, RR = 0.74 (95% CI 0.57- 0.96), and day 8 RR = 0.7 (95% CI 0.51 - 0.96). Adjusted for all co-variates non-significant decrease in unscheduled return visits: day 14 RR 0.8 (95% Ci 0.55 to 1.15); day 8 RR 0.7 (95% CI 0.44 to 1.10). No significant difference in unscheduled admission within 14 days of ED discharge (OR 0.92, 95% CI 0.59 to 1.42).	Pre/post design. Patients not blinded. Small sample size with complete data thus potentially affecting ability to reach significance.

**Table 2:** Summary table of studies describing interventions based in acute care settings

Study (Author, Year, Country)	Target population	Study Design & setting	Intervention	Control	Outcomes	Results/Main Findings	Quality
Interventions in emergency observation and assessment wards							
Emergency Department Observation or Decision units							
Storrow et al., 2005, USA [27]	Patients ≥18 undergoing evaluation for suspected heart failure (HF) exacerbation. Only those classified as low-to-moderate-risk eligible for inclusion.	Observational sequential cohort study (pilot study). Observation unit established in ED.	Observation unit available to treating physician to use in treatment (n = 28).	Heart failure standard care without observation unit available to treating physicians (n = 36).	Repeat visits to ED and readmission with primary complaint of HF all within 30 days.	No significant difference in hospital readmission rates (p = 0.538).	Potential for enrolment bias by treating physician. Observational study.
Foo et al., 2012, Singapore [33]	Patients ≥ 65 in the emergency department observation unit (EDOU). Thirteen conditions were accepted into the EDOU. Patients excluded if had poor premorbid condition, nursing home resident or those admitted to inpatient ward from EDOU.	Before/after prospective study. Emergency department observation unit.	Geriatric assessment and intervention in the EDOU prior to discharge by emergency nurse trained in geriatric care; exploring the patient’s medical, social and functional status (with referral to physiotherapist, appropriate community or social care services or GP if required) (n = 315).	Historical controls received usual EDOU care (n = 172).	Unscheduled ED re-attendance and hospitalisation at 3, 6, 9 and 12 months.	Significant reduction in ED re-attendance at 3, 6, 9 and 12 months: overall reduction of 41% (adjusted IRR 0.59, 95% CI 0.48–0. 71) at 12 months. Hospitalisation rates significantly reduced at 3, 6, 9 and 12 months: overall reduction of 36% (adjusted IRR 0.64, 95% CI 0.51–0.79)	Before/after design. Possible that recruitment process favoured a positive outcome.
Schull et al., 2012, Canada [31]	Data suggest adult patients attending ED.	Retrospective analysis of the difference in median ED LOS and admission rates among all ED visits after versus before CDU implementation at pilot-CDU and control sites. First 18 months of CDU operation compared with 1 year baseline period prior to CDU. Pilot-CDU sites (7). All CDUs within or next to ED.	7 Pilot CDU sites. Staffing models varied by site. Variation in CDU protocols. Number of beds varied by CDU site (n = 455, 942).	9 EDs without a CDU. ED had been unsuccessful in applying for pilot-CDU funding (n = 1,172,305).	Admission rates, ED revisit rates (after 48 hours, 72 hours, 7 days and 30 days) and ED length of stay.	Small decrease in hospital admission rate high-acuity patients: −0.8% (−1.5% to −0.03%) and moderate-acuity patients: −0.6% (−1.1% to −0.2%). No changes in ED revisit rates. 4% of ED patients admitted to CDUs.	Only 4% of ED patients admitted to CDUs. No mention of target population. Difficult to see efficiency gains. Pilot study. Missing retrospective data. Different sites had different protocols, staffing etc.
Conroy et al., 2014, UK [15]	Patients presenting to ED ≥16 years.	Pre-post cohort before and after establishment of Emergency Frailty Unit (EFU). Emergency Frailty Unit (EFU).	Comprehensive geriatric assessment in the EFU. Unit included input of acute medical consultant and later full geriatrician coverage (08 h00 – 18 h00, 7 days a week). Intervention moved to geriatrician integrated assessment with focus on patients identified for discharge and improvement of pathways to community. (n = 110, 517).	Usual care (model of care using Emergency Decision Unit without specialist geriatric input) situated in ED (n = 109, 994).	Primary: ED conversion rate (admission avoidance). Secondary: readmissions following attendance at 7,30 and 90 days.	Admissions (ED conversion rate) for patients >85 years fell from 69.6% (control) to 61.2% (intervention) (95% CI: 66.0– 73.1%) in the control period, p < 0.001. RR 0.88 (95% CI 0.81-0.95) Readmission rates fell across all age groups comparing intervention and control groups. Readmission risk ratio for those 85+ 0.77 (95% CI: 0.63–0.93) for 90 day readmissions. ED attendance increased in older people (65+) over the study period. ED attendance decreased for 16–64 year olds over study period.	No concurrent control group therefore causal effect difficult to establish.
Emergency Department Assessment units/wards							
Li et al., 2010, Australia [11]	All general medical patients presenting to ED.	Retrospective before and after study. Before and after the establishment of an AAU. Acute assessment unit at a University teaching hospital	Establishment of an acute assessment unit. Remit to receive adult patients who were not clinically appropriate for sub-speciality medical unit or for a surgical service (n = 3992).	ED patients requiring admission either referred to subspecialty service or to an ‘on-take’ medical team of the day (n = 2652).	Rate of unplanned readmissions within 7 and 28 days.	No change in the rates of unplanned readmissions within 7 and 28 days. At 7 days 3.8% (pre AAU) vs 3.7% (post AAU). At 28 days 8.7% (pre AAU) and 8% (post AAU) (p = 0.80).	Observational, uncontrolled study. May be affected by unknown bias and confounders.
Roberts et al., 2010, UK (Northern Ireland) [19]	Patients ≥16 with probable medical conditions, likely to be admitted through processes of standard ED care, but may potentially have been managed by a GP or as an out-patient following senior review.	Retrospective cohort. CDU cohort compared to three age-stratified, historical cohorts from same clinical centre. Clinical decision unit (CDU) located within ED. Pilot CDU (3 beds). Staffed by middle-grade physician and experienced nurses.	All patients who participated in the pilot CDU were included in the study cohort (n = 854). Most patients in the CDU group sourced from the ‘Major’ area in the ED.	Three comparison cohorts chosen from the preceding 3 years −2003, 2004 & 2005. These patients identified as those classified as ‘Medical’ by triage nurse a group most likely to have been diverted to the ‘Major area’. These were selected on an age-stratified basis, using the study cohort as the template (n = 854 for each cohort).	30-day unplanned re-attendance rate for those not hospitalized, and monthly medical admission figures.	Significant difference found in admission patterns of the different cohorts. Approximately 511 (59.8%, 95% CI: 56.5-63.1%) to 560 (65.6%, 95% CI: 62.3-68.7%) admitted in comparison group vs 186 (21.8%, 95% CI: 19.1-24.7%) in CDU (intervention) group P < 0.05. A greater proportion of patients from CDU had unplanned re-attendances 11.8% (95% CI: 9.5-14.5%) compared with between 4.4% (95% I 2.6-7.4%) and 7.5% (95% CI: 5.1-11%). P > 0.05 NOT SIGNIFICANT for all cohorts. Modestly significant compared to 2003 and 2004 cohorts.	Historical cohorts can’t exclude residual confounding.
Rogers et al., 2011, UK [20]	Adults (≥18 years). All GP referrals with a view to medical admission, but that are possibly avoidable, included either in MAU and/or by the GP support unit (GPSU).	Before and after study. Observational analysis. Analysis of number of patients referred and admitted to an MAU during a 6 month intervention period compared to control period. Emergency MAU in one acute hospital.	Team of GPs working near emergency MAU (GP support unit). All GP emergency medical referrals made between 10:00–19:00 on weekdays discussed with GPSU rather than MAU.	6 months prior to GPSU in situ.	Number of patients referred and admitted on week days by different modes (A&E, GP and GP via A&E). Total number of referrals and admissions.	Mean number of GP referrals to MAU per day decreased by 1.55 (−2.45 to −0.51). Non-significant decrease in mean number admitted to hospital per day from MAU 0.48 (−1.39 to 0.44). GP admissions not targeted through GPSU increased by 3.99 per day (2.64 to 5.33). Modest reduction in GP admissions to MAU but no reduction in number of GP admissions to hospital wards.	Before and after design.
Ong et al., 2012, Australia [13]	Patients ≥65 years. Diagnosis groups: falls and gait disorder, COPD, other major respiratory diseases, cellulitis. Target patients those requiring a short stay admission with potential discharge within 48 hr and sub-acute patients with multiple-comorbidities.	Retrospective case–control. Medical files of patients reviewed. MAU and general medical ward. MAU “Assess and manage undifferentiated patients for 36-48 h before transfer to medical ward or discharge home.”	Patients admitted to Medical Assessment Unit (MAU) before ED assessment completed and allied health review initiated when required (n = 47).	Patients admitted to General medical wards through standard ED assessment and management (n = 42).	Hospital readmissions in 1 month	No significant difference in readmission rate. Readmissions within 1 month similar in both groups (4.2% MAU) and (4.8% non-MAU group). MAU group shorter ED LOS (4.9 + − 3 h vs 6.5 + − 2.8 h, p = 0.012).	Small sample size and short duration of study. Retrospective design. Confounding.
Hospitalised patients enrolled into study within 72 hours of admission							
Enhanced care/discharge planning							
Koehler et al., 2009, USA [25]	High-risk elderly medical in-patients. ≥70 years, use of ≥ 5 medications regularly, ≥ 3 chronic comorbid conditions, require assistance with ≥1 ADL (predisposed to unplanned readmission or ED re-attendance). Patients enrolled within 72 hours of admission and likely to be able to be discharged home.	RCT – pilot. Medical in-patients. 2 medical units.	Intensive patient-centred educational program (by ‘highly experienced’ research staff) starting no later than 24 hours after enrolment. Medication counselling/reconciliation, condition specific education/enhanced discharge planning by a care coordinator, and phone follow-up (n = 20).	Usual care (n = 21).	Unplanned hospital readmission or ED visitation at 30 and 60 days post discharge.	0-30 day post discharge readmission/ED visit rates lower in intervention group (n = 2 vs 8) p = 0.03. No difference in 31–60 day readmission/ED visits. Longer time to first visit event in intervention vs usual care group (36.2 versus 15.7 days p = 0.05).	Small sample size. Incomplete blinding. Pilot study.
Lisby et al., 2010, Denmark [34]	Patients ≥70 years, in acute internal medicine ward and taking at least one drug daily with expected admission >24 hr.	RCT, non-blinded. Acute Internal medicine ward.	Clinical pharmacist conducted medication reviews and drug counselling after usual medication review in the ward. Medication history conferred to pharmacologist and medication changes recommended (n = 50). Intervention conducted within 24 hr of admission or by first-coming day of week.	Usual medication review in ward (n = 49). Usual medication review on admission (junior physician) and within 24 hr of admission by senior physician. Ward physicians not obliged to follow recommendations of routine medication review.	Number of emergency department visits. Readmissions.	No difference in ED visits Mean (95% CI) Intervention 0.1 (0.0-0.2) and control 0.1 (0.0 to 0.2). No significant difference in readmissions intervention 0.4 (0.3-0.6) and control 0.5 (0.3-0.7).	Possible contamination bias. Trial in one clinical setting and contamination bias could have optimized drug prescriptions in the control arm. Insufficient statistical power to detect a significant difference.
Bowles et al., 2014, USA [23]	Hospitalized patients aged ≥55 years. Study data collected within 24–48 hours of hospital admission.	Quasi-experimental study at one medical centre. 4 medical units at one urban hospital, “Primary practice setting”.	The Discharge Decision Support System (D2S2) used to assess patients within 24–48 hrs of admission. Results shared with case managers to alert them of patient’s risk status and to arrange referral for post-acute care where necessary (high-risk – refer and low-risk –do not refer) (n = 252).	Usual care. D2S2 completed but information not shared with case managers (n = 281).	Readmission outcomes at 30 and 60 days.	Percentage of high-risk patients readmitted by 30 and 60 days decreased by 6% and 9% respectively. Showing a 26% relative reduction in readmission of high-risk patients in pre and post intervention phases.	Two-phase study: additional interventions may have resulted in the changes seen. Limited to a single hospital - lacks generalizability.
Goldman et al.,2014 USA [24]	Hospitalized adults ≥55 years with anticipated discharge into community. Patients enrolled who had been admitted in the previous 24 hours.	RCT Safety-net hospital (provide care for patients at high risk of readmission.) Hospitalized adults (internal or family medicine, cardiology or neurology departments)	In-hospital, one-on-one, self-management disease-specific education by nurse within 24 hours of discharge (in preferred language). Telephone follow-up after discharge (on days 1 to 3 and 6 to 10). Patients had access to telephone support line – calls returned within 24 hours. On discharge patients received ‘After Hospital Care Plan’ booklet (n = 347).	Usual discharge care (n = 353).	ED visits or readmissions at 30, 90 and 180 days after discharge.	No statistically significant differences in ED visits or readmissions between intervention and control groups. HR (30 days) 1.26 95% CI; 0.89 to 1.78 (p = 0.19). HR (90 days) 1.21 95% CI 0.91 to 1.62 (p = 0.19). HR (180 days) 1.11 95% CI 0.86 to 1.43 (p = 0.44). ED VISITS (not hospitalised) 30 days HR 1.41 95% CI 0.81-2.44 (p = 0.22). 90 days HR 1.41 (0.88-2.24) (p = 0.15). 180 days HR 1.41 (0.97-2.06) (p = 0.07). Intervention group had greater proportion of patients with 2–5 ED visits.	Study lacked power due to lower than expected rates of readmission. Possible enhanced care given to’usual care patients’. Single centre study.
Greening et al., 2014, UK [17]	Patients aged ≥40 admitted to hospital with an exacerbation of chronic respiratory disease. Patients randomised within 48 hours of hospital admission.	RCT. An acute cardiorespiratory unit and an acute medical unit.	Early rehabilitation intervention started within 48 hours of admission and delivered by physiotherapists and nurses. Education and self-management package also part of intervention. Intervention lasted 6 weeks. Post discharge unsupervised home based program with telephone support at 48 hrs, two weeks and four weeks (n = 196).	Standard care from in-patient physiotherapist, dietician referral if necessary. Out-patient pulmonary rehabilitation offered three months after discharge (n = 193).	Readmission rate at 12 months. Readmissions for respiratory and other causes.	No significant difference in readmission rates between intervention and control groups (HR 1.1, 95% CI 0.86 to 1.43, p = 0.4).	Excluded patients with more than 5 admissions in the preceding 12 months.
Chronic disease specific interventions							
Kampan, 2006, Thailand [35]	Type 2 Diabetic patients hospitalized with hypoglycaemia.	RCT One hospital	Counselling and clinical pathway for treatment of hypoglycaemia. Assessment and treatment within the first 3 consecutive days of hospitalization (n = 33).	Conventional treatment for hypoglycaemia (n = 32).	Readmissions with recurrent hypoglycaemia at 1 and 3 months.	Significant decrease in readmissions with hypoglycaemia at 1 and 3 months in intervention compared to control group (6.06% intervention vs 34.38% control group; p = 0.036).	Insufficient evidence regarding randomisation. Study staff aware of treatment allocation. Likely not blind to intervention.

Studies that were exclusively of children attending the ED were excluded. The included evidence was restricted to countries within The Organisation for Economic Co-operation and Development (OECD) to ensure relative health system comparability to the United Kingdom (UK) National Health Service (NHS) and needed to be an English language publication.

Two authors (AS and EH) conducted the database searches. Two reviewers (SHC and EH) undertook an initial title and abstract screen, using the review’s inclusion and exclusion criteria. A third reviewer (CO) undertook a random screen of 10% of these and any discrepancies were resolved through discussion with this third reviewer. The full texts of all potentially eligible papers were reviewed by two reviewers (CO and SHC) and the final list of papers was agreed by consensus.

### Data extraction

Three reviewers (CO, SHC and EH) extracted data into standardised data extraction forms. The following data was extracted for each study: standard bibliographic information; target population; study setting; study design; description of the intervention; description of the control; reported outcomes and relevant study findings. Information on the study quality was also extracted. A 10% sample of papers was cross-checked between reviewers to ensure accurate data extraction.

### Assessment of quality

Quality assessment of each paper was undertaken by the reviewers extracting the data. This assessment included a review of each paper according to the Critical Appraisal Skills Programme (CASP) checklist appropriate for the study design being reported [6]. The assessment of quality was further informed by the limitations as reported by the authors of the studies under review.

### Data synthesis

Data for this review was extracted into tabular form and used to inform the narrative review. The considerable heterogeneity of the included studies did not lend itself to the consideration of a meta-analysis.

## Results

### Study selection

**Fig. 1:**
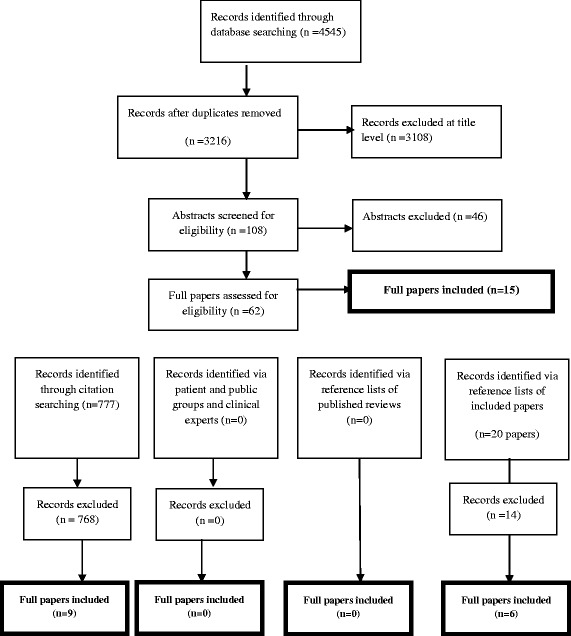
Flow chart of study identification

The database search for this review identified 4545 references; after removal of duplicate references 3216 unique references were identified. Of these, the full texts of 62 papers were examined and 15 papers included. Fifteen additional papers were included from those identified through additional search strategies – nine papers from citation searching and six were identified from the reference lists of included papers. Having sent the final list of included papers to experts within the field of emergency and acute medicine, no additional papers were identified. In total 30 papers met the inclusion criteria and are included in this review. Figure 1 details the process of study identification and final inclusion.

### Characteristics of the reviewed studies

The thirty papers included in this study all describe studies that enrolled or conducted an intervention with patients on the ‘journey’ from ED arrival to in-patient ward admission (within 72 h of admission). Of these studies 19 were intervention studies (14 randomised controlled trials (RCTs)) the remaining 11 were controlled observational studies. The majority (8) of the papers were conducted in Australia [7–14]. Seven studies were conducted in the UK [15–21], six in the USA [22–27], four in Canada [28–31], two in Singapore [32, 33] and one each in: Denmark [34], Thailand [35] and China [36]. Study sample sizes ranged from 41 patients (pilot RCT) to 1, 628, 247 patient records in a retrospective analysis.

Sixteen studies were set in the ED and the remaining 14 studies were conducted in an observation unit, acute assessment ward or in-patient ward. The study characteristics as well as the principle findings of each study are summarized in Tables 1 (ED) and 2 (Acute care). Emergency department interventions were pragmatically categorised into three groups, according to the stage of the patient’s journey during which the intervention took place. These categorizations included: interventions that took place during the ED attendance; interventions which were initiated in the ED and included a component in the community and post-discharge interventions which were initiated in the ED.

In order to classify the interventions according to where they occurred on the patient journey after ED presentation the following definitions, as proposed by Cooke et al., [37] have been used. Papers were classified according to the name given to the study setting by the author or the length of time the patient was anticipated to be in a particular setting as reported in the study.

#### Assessment unit/ward

An area where emergency patients are assessed and initial management undertaken by inpatient hospital teams. The patient is only in this area while early assessment is made, for example, up to 12 h and is then moved to another ward.

#### Observation ward

An area where patients can be observed or have early investigation/management within the A&E [Accident and Emergency] department. Patients are admitted to this area with an expectation of discharge within 24 h.

#### Admission ward

A ward to which people are admitted after clinical assessment for their continuing management [37].

### ED results

Of the 16 studies based in the ED, two studies reported on interventions that took place during ED presentation, ten were studies that were initiated in the ED and include a component in the community and the remaining four studies were post-discharge interventions started in the ED. Of these 16 studies, 13 interventions targeted patients 65 years or older [7–10, 12, 16, 21, 22, 26, 28–30, 32], two included all adults over 18 years [14, 18] and one reported on both adults and children attending an A&E [36]. Fifteen papers set in the ED measured ED attendance, six of these also measured hospital admission (including readmission) as outcomes; one paper reported hospital admission only.

#### ED based interventions (occurring during ED attendance)

Two studies described interventions that took place during the patient’s time in the ED [12, 14]. One of these studies, which was non-randomised, involved the introduction of a specialist aged care pharmacist to provide medication reconciliation and review as well as patient education to elderly patients [12]. This study was not effective in reducing ED re-attendance but showed a possible reduction in admission rates for the intervention group [12]. However, this result was not tested for significance. The other study, set in the ED, was a randomised controlled trial, of patient centred education for asthmatic patients [14]. Results from this study suggest that at 4 months there was no significant difference in ED attendance between the intervention and control groups [14]. However, after controlling for general practitioner (GP) attendance the intervention group had significantly fewer ED re-attendances [14].

#### ED initiated interventions which include a post-discharge community component

Ten studies initiated an intervention in the ED that involved a post-discharge community component [7–10, 16, 18, 21, 26, 30, 32]. Each intervention differed but could be grouped under the following headings (Table 1): comprehensive geriatric assessment; multi-factorial falls intervention or specialist nurse assessment. Nine out of these ten studies included patients over the age of 65 years. Of the ten studies in this setting, two were effective in improving their primary outcomes [9, 10], one of these was an RCT. The RCT had an intervention which involved comprehensive geriatric assessment over a four week period [9]. The other study provided specialist community nurse risk screening for elderly patients prior to discharge [10]. A further two studies initiated in the ED showed a paradoxical increase in intervention patients re-attending the ED [8, 30].

#### ED initiated post-discharge interventions

The third categorization included four studies where the intervention was initiated at ED discharge and included a component of follow up or monitoring post discharge [22, 28, 29, 36]. These included a study of an intervention that used personal emergency response systems and a further three that provided a nurse led telephone or telehealth post discharge intervention. One study, which adjusted for severity of patient illness, found a significant reduction in unscheduled return visits following discharge facilitated by a nurse discharge plan co-ordinator [28]. A further study paradoxically found that intervention patients were significantly more likely to return to the ED within 30 days of initial attendance [36].

### Acute care setting

#### Results

Within the acute care setting, four studies were conducted in observation wards or decision units [15, 27, 31, 33], where the patient is expected to be discharged within 24 h, and four were conducted in ED assessment units or wards [11, 13, 19, 20]. The remaining six papers describe studies where the patients were enrolled within 72 h of hospital admission [17, 23–25, 34, 35]. Nine of the studies within the acute care setting targeted adult patients [13, 17, 20, 23–25, 27, 33, 34] one study included patients from 16 years [15] and three studies included patients of any age meeting their other inclusion criteria [11, 31, 35]. All of the papers in the acute setting reported admission (including readmission) as an outcome, seven of these (50%) also reported ED attendance as an outcome.

#### ED observation or decision units

Of the studies set in observation wards or decision units, two evaluated complex interventions that involved geriatric assessment, multi-disciplinary team intervention and community referral and two evaluated the effectiveness of the unit/ward on the outcomes of interest. Two interventions, both before-after studies, were effective in reducing the review outcomes of interest: ED re-attendance [33] and hospital admissions (ED conversion rate) [15], one of these was also effective in reducing re-admissions [15]. Foo et al., [33], provided geriatric assessment and appropriate intervention in an emergency department observation unit with follow up referral where necessary. Conroy et al., [15], evaluated the establishment of an emergency frailty unit on patient admission and readmission.

#### ED assessment units/wards

The interventions that took place within an ED assessment unit either assessed the establishment of the unit [11, 13, 19] or assessed the impact of a general practitioner (GP) support unit within a medical assessment unit (MAU) [20]. One study, a retrospective cohort, showed a significant reduction in admissions in favour of the study group [19]. However, in this study a greater proportion of patients in the intervention group had an unplanned ED re-attendance [19].

#### Hospitalized patients enrolled within 72 hours of admission

The studies into which patients were enrolled within 72 h of hospital admission [17, 23–25, 34, 35] involved enhanced care or discharge planning [17, 23–25, 34] and one paper reported on a chronic disease specific intervention for Type 2 Diabetes [35]. All of the studies reported hospital readmissions as an outcome and three reported ED revisit rates [24, 25, 34]. Three studies showed a significant reduction in ED readmission [23, 25, 35]. One of these was an RCT which included Type 2 diabetic patients and offered counselling and a clinical pathway for the treatment of hypoglycaemia in comparison to usual care [35]. The second study which was also an RCT, but a pilot RCT, provided intensive patient-centred education for high-risk elderly medical in-patients [25]. The final study which showed effectiveness was a quasi-experimental design comparing a discharge decision support system to usual care [23].

## Discussion

This rapid evidence synthesis has found limited evidence of interventions along the patient journey through the ED that are effective in reducing hospital admission and/or ED attendance. This review provides a more in-depth review of the patient pathway from the ED to acute admission than a recent review which similarly found insufficient evidence to determine whether services in the ED reduced unplanned admissions [5]. The interventions included in this evidence synthesis are of varying complexity, often comprising a number of different components which may be unique to a particular study setting (such as assessment and discharge planning by different types of health professional, different discharge pathways and additional care). This means it is difficult to establish exactly which elements of an intervention are impacting on outcomes which affect the generalisability of study findings.

In addition, the nature of the health problems and severity of illness among patients in the included studies varied greatly and may impact on the degree to which the interventions were effective. The type of health problem, and severity of the presenting condition, plays a large role in determining whether or not a patient is eligible for an intervention; what the nature of this intervention is; and where this intervention occurs within the healthcare system. Some studies included all adult patients attending [11, 15, 31] while others risk stratified patients and only included those of low-moderate risk [27]; only those at high-risk [25] or those with potentially avoidable admissions [19, 20]. The selection of ‘high-risk’ patients or those with poor baseline health with a background of chronic illness may be a reason for lack of intervention effect if the underlying chronic conditions increase the risk of admission [8, 26]. In contrast Lee et al., [29], did not restrict their patient sample to ‘high-risk’ patients and suggest that had they chosen the group most likely to benefit from the intervention a positive intervention effect may have been seen.

What is apparent from the study findings is that high quality, prospective research is needed looking at complex interventions within the ED and acute care setting to reduce ED attendance or unplanned admission. In developing interventions researchers need to be guided by existing evidence regarding what may be effective; should ideally use randomised control trial methodology and include a pilot phase [38]. Furthermore, the intervention should be evaluated using an appropriate choice of outcome measures that provide an adequate assessment of the success of the intervention. The successful interventions included in this review include a number of features that may have contributed to their effectiveness and these warrant further high quality research.

Firstly, the literature suggests that ED initiated interventions that include comprehensive assessment or screening and community follow-up or referral have aspects that may have contributed to their effectiveness. The majority of the included studies (19/30) targeted their interventions at adults >55 years highlighting the focus on elderly care patients. Three studies that were effective in reducing admissions all included elderly patients, involved assessment by a specialist nurse and provided further treatment and referrals to appropriate providers [9, 10, 28]. These studies suggest that assessment and management of older people at risk of admission can improve their health outcomes. Accurate identification of patients in need of community support by trained nurses and services with appropriate follow up care may be effective in reducing ED attendance and hospital admission rates. Despite the promise that these interventions hold, the findings are not supported by Mion et al., [26], who reported no statistically significant effect on overall service use rates. This study intervention may have been weakened by a lack of advance practice nurse involvement after follow up which makes comparison with other studies difficult.

Secondly, the results suggest that the qualifications and specialties of the assessing and treating team members may impact on service utilization outcomes. A specialist nurse rather than a triage nurse, used in the intervention by Hegney et al., [10], impacted positively on service utilization. Guttman et al., [28], support this idea. In their intervention, study nurses were selected for their expertise in nursing care and had a minimum of 5 years nursing experience within acute care. This is important as the complexity of discharges and the hurried discharge conditions often present in the ED may be beyond the scope of a primary ED nurse [28]. In addition, as well as the usual emergency physicians, the clinical leads in Conroy et al’s., [15], paper included geriatricians and emergency medicine nurses with additional training in geriatric syndromes and manual handling. Ensuring that team members were appropriately trained to manage and treat or refer patients appropriately may have contributed to the effectiveness of some of the included interventions.

A systematic review that looked at geriatric specific interventions on ED utilization found that the source of patients (ED, out-patient or home care setting) and the type of intervention impacted on the utilization rates [39]. Studies which recruited patients in the ED had little effect on ED utilization, partly, the authors believe, related to the limited follow-up duration for patients discharge from the ED and the difficulty in facilitating appropriate community follow-up and referrals from the ED [39]. Given that only two of the 16 ED based studies in our review were effective in reducing ED attendances or admissions (and a further two on sub-group analysis) may suggest that intervention location may have impacted on these study results. It may be the case that interventions should be trialled away from the time pressured environment of the ED and within observation or assessment wards to reduce unplanned admissions [37]. For patients discharged directly from the ED allowing sufficient time to plan the discharge care of patients may reduce the proportion of unscheduled ED return visits.

Observation and assessment wards, allow a greater length of time to assess and manage patients compared to the ED, and this additional time may have contributed to the positive findings of interventions to prevent re-attendance and readmission in these settings. Older patients who receive comprehensive geriatric assessment, allied health intervention and referral prior to discharge, from an observation unit have decreased ED utilization [15, 33]. Allowing a greater length of time to assess and manage patients enables complaints, other than the primary complaint, to be addressed and these healthcare needs met resulting in reduced ED re-attendance and hospitalisation [33]. As it is not possible to provide comprehensive geriatric assessment to all patients, and for many this would be unnecessary, it is important that these interventions are targeted to high-risk patients [33].

Lastly, patient centred education within the ED may offer promise for specific chronic diseases. The results from the study by Smith et al., [14], found no significant difference in ED attendance rates although, after controlling for GP attendances, the intervention group had significantly fewer re-attendances. Educating patients according to their specific needs, guided by a curriculum, may be useful in reducing re-attendances to the ED as their healthcare needs are met. This finding is echoed in a Cochrane review that summarises education interventions for asthma in the ED which also suggests that hospital readmissions may be reduced through education interventions for asthmatics although the generalisability of the findings need to be confirmed in larger, multi-centred trials [40].

The interventions initiated within 72 h of patient admission have aspects that are similar to the above findings. Interventions that involved patient education, enhanced discharge and included patient follow up after discharge have been shown to decrease readmission and ED visits [25]. In addition, when high-risk patients are identified and their needs are met, including sufficient time to work with patients and families to agree a workable care plan, readmission rates have been seen to decrease [23].

Interventions that targeted specific chronic conditions were limited to four studies. The target populations included patients with: asthma [14], epilepsy [18], heart failure [27] and Type 2 diabetes [35]. The heterogeneity of the patient groups and the interventions precludes making meaningful statements about what is effective in chronic disease management. Patient education and specific clinical pathways require further research in the acute care setting.

It is also important to discuss the paradoxical increase in ED re-attendance and hospital admission that is evident in some of the included studies. Three ED based interventions had this effect [8, 30, 36]. The reasons for this paradoxical increase may be that greater assessment and screening of patients sensitizes patients to health problems and motivates them to seek healthcare and access further services [36]. McCusker et al., [30], also suggest that this increase may be as a result of lack of access to primary care services which is a known predictor for increased ED utilization. These findings have also been seen in a systematic review which concludes that while ED based interventions may show promise they can have the unintended consequence of increased demand on these services [41].

The interventions included in this study can be considered as complex interventions, which include several components [42]. It is acknowledged that interventions classified as ineffective in this review does not necessarily mean that the intervention was ineffective but the findings may be as a result of process failures, how the intervention was implemented or whether the follow-up time was sufficient to provide an adequate assessment of the success or failure of an intervention [38]. Furthermore, many of the interventions included in this study had beneficial effects on other service related outcomes, for example: decreased hospital length of stay [11, 35] or increased contact with PHC following discharge [22]. These outcomes are not covered by our review and it is acknowledged that these interventions may be effective in reducing other important outcomes.

### Limitations

The studies included in this rapid review were carried out in a variety of national settings with heterogeneous study designs and using different outcome measures and this limited our ability to synthesise the results of individual studies.

As this was a rapid review we did not score the quality of each individual included paper but took into account the limitations described by each author. The limitations of the papers were considered and these included non-randomised studies or before and after design cohort studies which are more susceptible to certain bias than RCTs, such as selection bias [7, 9, 12]. Without evidence from randomised controlled trials, confounding and other methodological flaws cannot be discounted in evaluating the findings.

As this was a rapid review, with limited time frame, we did not attempt to identify all relevant evidence through an exhaustive search. Through a well thought out and devised search strategy we aimed to identify the key evidence of most relevance to our review question.

## Conclusions

In the UK, emergency department attendances and emergency hospital admissions are continuing to increase. As the population ages medical admissions are also becoming increasingly complex as patients live longer with chronic medical conditions [1]. This review looked at the current evidence on interventions that reduce emergency hospital admissions and emergency department attendances with the aim of informing the design of new interventions to decrease service utilization.

**Fig. 2:**
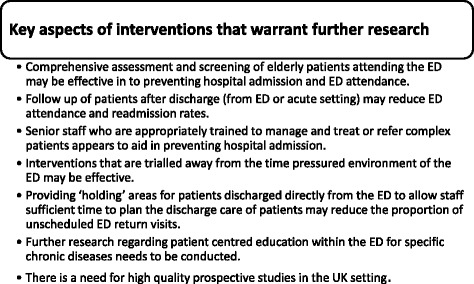
Key aspects of interventions, identified in rapid review, that warrant future research

A number of findings from this review (shown in Fig. 2) may be helpful in designing future interventions. Firstly, there is a need for high quality, prospective studies within the UK setting. Interventions targeted at high-risk patients, particularly the elderly, may reduce ED utilization and is worthy of future research. The development of future interventions should consider elements of interventions included in this review that were successful and may include: delivery by appropriately trained personnel and in an environment that ensures adequate time in which to assess and manage patients appropriately these elements may help to reduce the number of emergency admissions and the proportion of unscheduled return visits to the ED.

## Abbreviations

A&E: Accident and Emergency
CASP: Critical Appraisal Skills Programme
ED: Emergency Department
GP: General practitioner
MAU: Medical assessment unit
NHS: National Health Service
OECD: Organisation for Economic Co-operation and Development
RCT: Randomised controlled trial
UK: United Kingdom
